# *FGF* gene expression in injured tendons as a prognostic biomarker of 1-year patient outcome after Achilles tendon repair

**DOI:** 10.1186/s40634-021-00335-0

**Published:** 2021-03-11

**Authors:** Junyu Chen, Joel Svensson, Carl-Johan Sundberg, Aisha Siddiqah Ahmed, Paul W. Ackermann

**Affiliations:** 1 Department of Molecular Medicine and Surgery, Karolinska Institutet, 171 76 Stockholm, Sweden; 2 Department of Physiology & Pharmacology, Karolinska Institutet, Stockholm, Sweden; 3 Department of Learning, Informatics, Management and Ethics, Karolinska Institutet, Stockholm, Sweden

**Keywords:** Achilles tendon rupture, Patient outcome, Biomarkers, mRNA expression, immunohistochemistry

## Abstract

**Purpose:**

Healing outcome after Achilles Tendon Rupture (ATR) is variable and unsatisfactory. Many ATR patients still exhibit pain, functional deficits and limitations in walking one-year post-surgery. The present study was designed to investigate the association between the expression of healing biomarkers and patient outcome after ATR.

**Methods:**

Tendon biopsies were collected from 25 ATR patients during surgery. At 1-year post surgery, all patients completed questionnaires; Achilles tendon Total Rupture Score (ATRS) and Foot and Ankle Outcome Score (FAOS), and were tested for functional outcomes by heel-rise test. In biopsies, *FGF*, *COL III*, *FN*, *COL I* and *MMP-9* mRNA levels were assessed by quantitative RT-PCR while protein expression was studied by immunohistochemistry (IHC).

**Results:**

Our analysis confirmed the presence of *FGF*, *COL III*, *FN*, *COL I* and *MMP-9* at mRNA and protein levels in tendon biopsies. *FGF* gene expression associated positively with improved total ATRS and better functional outcomes. Additionally, *FGF* mRNA levels were associated with less pain, less running limitations and less loss in physical activity. In addition, higher *COL III* mRNA expression was associated with more tendon strength.

**Conclusion:**

Our findings indicate that *FGF* gene expression is associated with improved patient-reported outcome. FGF expression in surgical biopsies could potentially be used to assist the prognostic evaluation of patient outcome and may be used as a predictor for healing. However, further studies are needed to evaluate the role of FGF in Achilles tendon healing.

**Level of evidence:**

II

## Introduction

Achilles tendon rupture (ATR) is a common injury which is increasing in incidence worldwide [21, 22, 32]. Patient outcomes are variable and patients are often plagued by pain and limitations in physical activities [29, 32, 37]. One important reason for the difficulties in improving the treatment of ATR is the lack of functional biomarkers which can be used as predictors of tendon healing.

Achilles tendon repair is a complex process which involves variety of cytokines, growth factors, proteases and collagens [38]. Growth factors released from blood-derived cells at the site of injury activate tissue-derived cells such as fibroblasts to start the production of extracellular matrix (ECM) proteins and proteases. Fibroblast growth factor (FGF), an inflammatory mediator released by blood-derived cells, is crucial for the initiation of healing processes in tendons mainly by stimulating proliferation of fibroblasts [30]. Moreover, FGF plays an important role in the synthesis of ECM protein and formation of granulation tissue in injured tendon [17]. However, the association between FGF and ATR healing as measured by patient-reported outcome has, to the best of our knowledge, not been studied before.

ECM deposition by fibroblasts especially during the early healing is rich in collagen III (COL III) [28], which is replaced by collagen I (COL I) during the later healing phases. The degradation of COL III and remodeling into COL I is performed by matrix metalloproteinases (MMPs), especially MMP-2 and -9 [9, 12, 14, 18]. Another protein, fibronectin (FN), has been reported to play crucial roles in tendon healing by arranging the matrix fibrils [6, 12]. In addition, FN is reported to facilitate fibroblast adhesion, growth and migration in healing tendon [6]. The relationship, however, between collagens, proteases, FN and patient outcome after ATR healing is still unknown.

The aim of the study was to find potential biomarkers of tendon healing. For this purpose, the expression of FGF, COL III, FN, COL I and MMP-9 both at the mRNA and protein levels was studied in tendon biopsies collected from ATR patients and further the association between the expression of biomarkers and patient’s outcome at 1-year post surgery was investigated. We hypothesized that the expression of healing biomarkers influences patient’s long-term outcome and can be used as predictors of healing after ATR.

## Materials and methods

This study was conducted with ethical approval from the Regional Ethical Review Committee in Stockholm, Sweden (Reference no. 2009/2079–31/2, 2013/1791–31/3). All participants provided written informed consent.

### Study design

This is a retrospective study of twenty-five patients suffering with acute Achilles tendon rupture (ATR) who underwent reconstruction surgery at the local hospital. The majority of the ATR-cases, 90%, were sports-related. During the surgery, tendon biopsies were taken from the ruptured and the intact area and stored at minus 80^o^ C for future analysis. Patients were randomly selected from two patient cohorts who participated in randomized control trials. The patient outcome was evaluated 1-year postoperatively by using validated questionnaires; Achilles tendon Total Rupture Score (ATRS) and Foot and Ankle Outcome Score (FAOS). The functional outcomes were evaluated by using the heel-rise test (HRT).

### Inclusion and exclusion criteria for the patients

The inclusion criteria were; patients diagnosed with acute Achilles tendon rupture at the Karolinska University Hospital. The exclusion criteria was; 1) unable to give verbal and/or written consent for participating in the study; 2) currently treated with anticoagulants; 3) allergic to contrast liquid; 4) planned follow up on other hospital than Karolinska University Hospital Solna; 5) unable to follow instructions; 6) patients suffering from renal failure; 7) patients with symptomatic chronic heart failure; 8) patients with thromboflebitis or known coagulation disorder; 9) patients had received other surgery during the month before tendon rupture; 10) patients with known malignancy or pregnancy.

### Surgical and biopsy procedure

Local anesthetic was administered (20 ml of Marcain® and adrenalin 5 mg/ml, AstraZeneca, London, UK) in the dermis, subcutis and peritendinous space prior to surgery. The patients were then placed in prone position and a medial incision was made through the skin, fascia cruris and paratenon. The rupture was located by the surgeon and a 10 mm Achilles tendon biopsy was taken from the ruptured area, another 10 mm biopsy was taken 3-4 cm away from the rupture side as a control, from a visibly intact tendon area.

A modified Kessler suture with two 1–0 polydioxanone (Ethicon, Somerville, New Jersey, USA) sutures, was used to bring the tendon ends together. The paratenon and fascia cruris were then closed with 3–0 Vicryl (Ethicon, Somerville, New Jersey, USA) and the skin was sutured with 3–0 Ethilon (Ethicon, Somerville, New Jersey, USA). The same anesthetic and surgical techniques were used for all patient using a predefined study protocol.

### Post-operative treatment

All patients were prescribed paracetamol 500 mg or codeine 30 mg for administration if necessary, one to two pills at a maximum of four times per day. Patient included participated in two randomized trials evaluating post-operative treatments the first two post-operative weeks. None of the different post-operative treatments altered patient outcome at 1-year post surgery [1, 4, 10]. The first study evaluated treatment in a below-knee plaster cast for 2 weeks compared to orthosis and adjuvant intermittent pneumatic compression [1]. The other study evaluated treatment in a below-knee plaster cast for 2 weeks compared to early weight bearing in an orthosis [4]. The remaining 4 weeks of immobilization all patients were prescribed full weight bearing in an orthosis.

### Patient reported outcomes

Patient-reported outcomes were collected by using the ATRS and FAOS one-year post-operation during the follow up. ATRS consists of 10 sub-scales: strength in tendon, tiredness in the tendon, stiffness in tendon, pain in tendon, limitations in activity of daily life (ADL), limitation in uneven surface, stairs, running, jumping and loss in physical work [30]. Each sub-scale ranges from 0 to 10 where 0 = worst and 10 = best outcome with no limitation. The maximum ATRS is 100, and a score higher than 80 was regarded as good subjective outcome.

FAOS consist of 5 categories: Pain, Symptoms, Activities of Daily Living, Sport and Recreational activities and Foot-and Ankle-Related Quality of Life [34]. Each category ranges from 0 to 100 where 0 = worst and 100 = best outcome, and a score higher than 80 was regarded as good subjective outcome.

### Functional outcome

Functional outcomes were evaluated by heel-rise test (HRT) at 1-year post-surgery. HRT is a validated test [25, 36], which has been used in previous studies [2, 5, 33] to show the outcome of strength and endurance of the affected gastrocnemius-soleus complex. The HRT was performed on one leg with the patient standing on a box with 10°incline. The speed was set to 30 heel-rises/min using a metronome. Patients were instructed to perform as high heel-rises as possible and as many heel-rise repetitions as possible. The test was terminated when the patient stopped or could not maintain the frequency. All the results, including the number of heel-rises, the height of every single heel-rise, the total work in joules (total distance × body weight), the time and the power (work/time) were recorded for analysis. The Limb Symmetry Index (LSI) was used to show the ratio between injured and contralateral uninjured leg and results are presented in percentage (injured/contralateral).

### mRNA extraction and tissue homogenization

RNA was extracted from injured and intact tendon tissues collected from 20 subjects as described previously [31]. Briefly, the tissues were cut into small pieces at − 20 °C, put into pre-cooled tubes with steal beads containing 1 ml of tri-reagent (Sigma, Stockholm, Sweden) and immediately homogenized twice for 30 s by using a bead homogenizer [10] that shakes the tubes vigorously and ensures full homogenization. After addition of chloroform, samples were centrifuged to split into an aquatic and an organic phase. The aquatic phase was separated and precipitated with isopropanol to obtain RNA as pellet. Glycogen was added for better visualization of RNA pellet, washed twice with ethanol, dried and re-dissolved in 10 μl of RNase-free water.

### mRNA purification and quantitative real time-PCR (qRT-PCR) analysis

The RNA quantification was determined using a Nanodrop ND-1000 spectrophotometer (Isogen Life Science, Sweden), and RNA quality was measured as the RNA quality index (RQI) using the Experion electrophoresis system (BioRad, Sweden). First-strand cDNA was synthesized from 50 ng of total RNA using a first-strand cDNA Synthesis Kit (Roche, Germany).

For the qRT-PCR-analysis, RT-qPCR was performed using TaqMan Gene Expression Assays (Applied Biosystems, Carlsbad, CA) with the GeneAmp 7500 Fast Sequence Detection system (Applied Biosystems, Carlsbad, CA). Specific primers for *FGF, COL III*, *FN*, *COL I*, and *MMP-9* (MWG Biotech, Ebersberg, Germany) were used to detect target genes (Table 1). Data was normalized by using *GAPDH* as the reference gene and ΔΔCt method was used for analysis.

**Table 1:** Primers sequences (nucleotide sequences) used during quantitative RT-PCR

Genes	Forward Primer	Reverse primer
*FGF*	TGACGGGGTCCGGGAGAAGA	ATAGCCAGGTAACGGTTAGCACACAC
*COL III*	CACGGAAACACTGGTGGACAGATT	ATGCCAGCTGCACATCAAGGAC
*FN*	TTTGCTCCTGCACATGCTTT	TAGTGCCTTCGGGACTGGGTTC
*COL I*	GGCAACAGCCGCTTCACCTAC	GCGGGAGGACTTGGTGGTTTT
*MMP-9*	AGCGAGGTGGACCGGATGTT	AGAAGCGGTCCTGGCAGAAATAG
*GAPDH*	CCTCCTGCACCACCAACTGCTT	GAGGGGCCATCCACAGTCTTCT

### Histology

#### Fixation and staining

Intact and injured tendon tissues from 5 subjects were fixed in Zamboni’s fixative consisting of 4% paraformaldehyde in 0.2 mol/L Sörensen phosphate buffer, pH 7.3, containing 0.2% picric acid at 4 °C for 3–4 days. Tissues were then soaked in 20% sucrose in 0.1 mol/L Sörensen phosphate buffer, pH 7.2, containing sodium azide and bacitracin (Sigma Chemicals, St. Louis, MO, USA). Subsequently, Tissues were sectioned using a Leitz® 1720 cryostat (Ernst Leitz, Wetzlar, Germany) to a section thickness of 7 μm and mounted on SuperFrost/Plus slides. For each slide, 2 sections; one from the injured and another from the uninjured were mounted. All the sections were stored at − 20 °C until staining.

Sections were stained with hematoxylin and eosin (H&E) and Sirius red following standard procedures. Images were captured by a video camera (DEI 750; Optronics Engineering, Goleto, CA) attached to the microscope and stored in a computer.

### Immunohistochemistry (IHC)

Sections were marked with pap-pen and soaked in 1% PBS for 5 min before incubation with 1% H_2_O_2_ for 30 min. Sections were then blocked with 2% goat or horse serum, washed with 1% PBS for 3 × 5 min and incubated with Avidin and Biotin before overnight incubation with antibodies against FGF, COL III, FN, COL I and MMP-9 at room temperature. Sections were washed with 1% PBS for 3 × 5 min before incubating with 100 μl secondary antibodies (1:250, PBS-0.1% BSA) for 40 min. Secondary antibody, horse anti mouse was used for MMP-9, FN, FGF while for COL I and III, goat anti rabbit antibody was used. Slides were washed with 1% PBS for 3 × 5 min and incubated with 100 μl of ABC solution (Vector Laboratories, Inc. Burlingame, CA, USA), stained with DAB solution (Vector Laboratories, Inc. Burlingame, CA, USA) and counterstained with Hematoxylin (Vector Laboratories, Inc. Burlingame, CA, USA). This step was followed by dehydration in 70%, 96% and 100% alcohol and with the Xylene before mounting with pertex. To demonstrate specificity of staining, primary antiserum was either omitted or replaced by IgG. All the images were saved by the computer connected to the light microscope (DEI 750; Optronics Engineering, Goleto, CA, USA).

### Statistical analysis

All data was analyzed using SPSS (IBM SPSS, version 24.0), and Graphpad Prism Software 8.0. Descriptive statistics (means ± SD) were used to summarize the variables. For normally distributed data Student’s *t*-test was applied to detect differences among groups while the Mann-Whitney U-test was used for non-normally distributed data. The significance of correlation was determined by Spearman correlation co-efficient and effects of age, gender, BMI and TTS were determined by partial correlation analysis. A *p-*value ≤0.05 was considered significant.

## Results

### Patient characteristics

The study included 25 patients with an average age 39.9 ± 7.4 years. Body mass index (BMI) was recorded as 25.6 ± 3.1 kg/m^2^ and time from injury to surgery (TTS) was 67.0 ± 29.1 h (Table 2).

**Table 2:** Basic characteristics of subjects and their clinical information

	Subscale	ATR Patients	^a^Residual symptom % (n/N)
Study subjects (N)		25	
Average Age (years ± SD)		39.9 ± 7.4	
Gender (Male: Female)		12:5	
BMI (kg/m^2^)		25.6 ± 3.1	
TTS (hours)		67.0 ± 29.1	
ATRS		80.4 ± 12.4	
	Strength	7.1 ± 1.8	100 (17/17)
	Tired	7.4 ± 2.3	82.4 (14/17)
	Stiffness	7.3 ± 2.1	82.4 (14/17)
	Pain	9.5 ± 0.8	35.3 (6/17)
	ADL	8.8 ± 1.7	58.8 (10/17)
	Surface	9.2 ± 1.3	35.3 (6/17)
	Stairs	8.8 ± 1.2	58.8 (10/17)
	Running	7.2 ± 2.4	76.5 (13/17)
	Jumping	6.1 ± 2.7	100 (17/17)
	Physical work	9.0 ± 1.4	52.9 (9/17)
FAOS	Pain	94.6 ± 7.4	52.9 (9/17)
	Symptom	87.9 ± 11.9	76.5 (13/17)
	ADL	97.1 ± 5.1	64.7 (11/17)
	Sport and Recreation	80.0 ± 14.8	88.2 (15/17)
	Foot-and Ankle-Related QOL	74.5 ± 15.4	82.4 (14/17)
HRT	LSI Power	83.1 ± 13.2%	88.2 (15/17)
	LSI Total work	75.5 ± 17.7%	88.2 (15/17)
	LSI Repetition	90.8 ± 20.3%	76.5 (13/17)
	LSI Average height	83.8 ± 12.4%	94.1 (16/17)

Demographic and clinical characteristics of ATR patients included in study. Data presented as mean ± Standard Deviation. (*BMI* Body mass index, *TTS* Time to surgery, *ATRS* Achilles tendon Total Rupture Scale (0–100, and 0–10 for each subscale, worst = 0), *FAOS* Foot and Ankle Outcome Score (0–100, worst = 0 for each catagory), *HRT* Heal Rise Test (0–100, worst = 0 for each catagory); *ADL* Activities of Daily Living, *QOL* Quality of Life, *LSI* Limb Symmetry Index) ^a^*Patients with residual symptoms* (0–100, worst = 100) *were categorized as ≤ 99 in ATRS, ≤ 9 in ATR sub-scales, ≤ 99 in FAOS and ≤ 99% in HRT ratio. n/N = number of patients/total number of study patients.*

### Patient-reported outcomes

Total mean ATRS was noted as 80.4 ± 12.4 at one-year post injury. ATRS subscales indicated that 35% of patients experienced pain to a small extent while more than 80% of the patients suffered from tiredness and stiffness in the tendon and experienced limitations in strength and jumping at 1-year post-surgery (Table 2).

FAOS indicated that 53% of the patients experienced a small degree of residual pain and more than 80% of the patients suffered of limitations in foot and ankle quality of life and in sports activities at 1-year post surgery (Table 2).

### Functional outcome

At 1-year post-surgery, more than 80% of the patients experienced functional deficits in the injured side compared to the contralateral intact side using the HRT (Table 2).

### Gene expression analysis

**Fig. 1:**
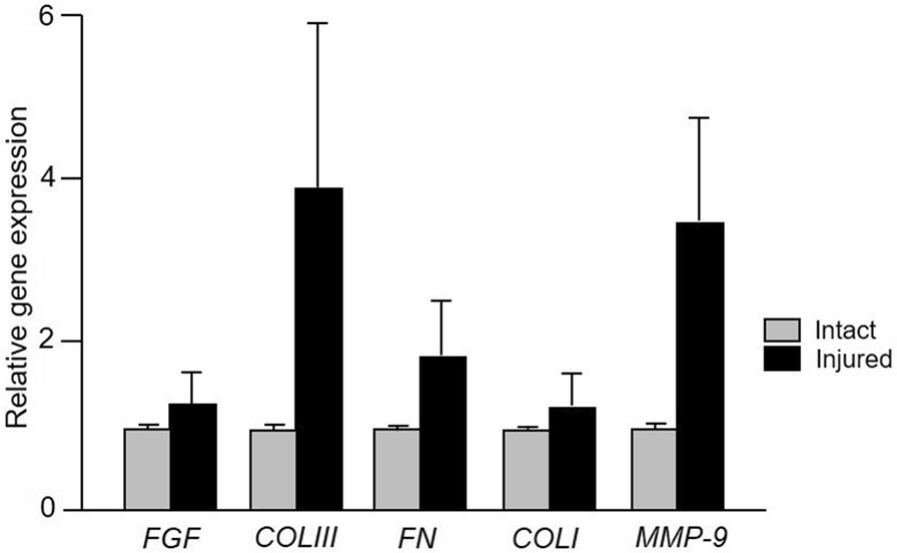
Relative gene expression of *Fibroblast growth factor* (*FGF*), *collagen III* (*COL III*), *fibronectin* (*FN*), *collagen I* (*COL I*) and *matrix metalloproteinase-9* (*MMP-9*) in biopsies collected from the injured and intact area of tendon. Values reported are mean ± SEM *n* = 12

Our quantitative RT-PCR analysis showed that all studied genes were expressed in measurable levels in tissues collected from both intact and injured areas of the ruptured tendon. The expression was higher in injured areas compared to the visibly intact areas of the ruptured tendon, however, no statistically significant difference in the mRNA expression was observed for any of the genes (Fig. 1). Due to technical or handling complications, detectable RNA could be obtained from 12/20 patients.

### Association between gene expression and patient reported outcome

**Fig. 2:**
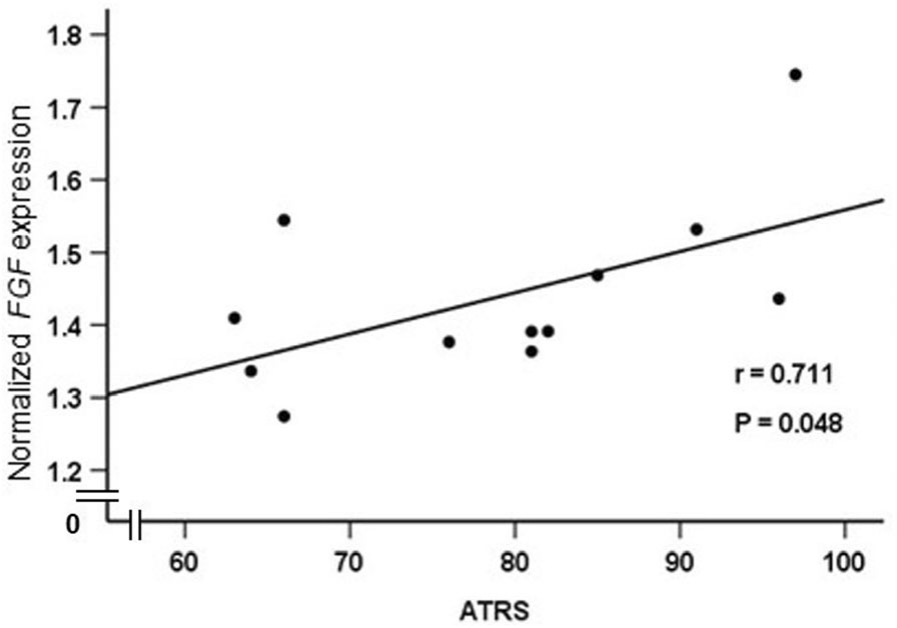
Correlation between *FGF* mRNA expression and total ATRS as measured by Spearman’s rank correlation coefficient. *FGF* mRNA expression was normalized and ATRS ranges from 0 to 100, with 100 = best outcome. *n* = 12

**Fig. 3:**
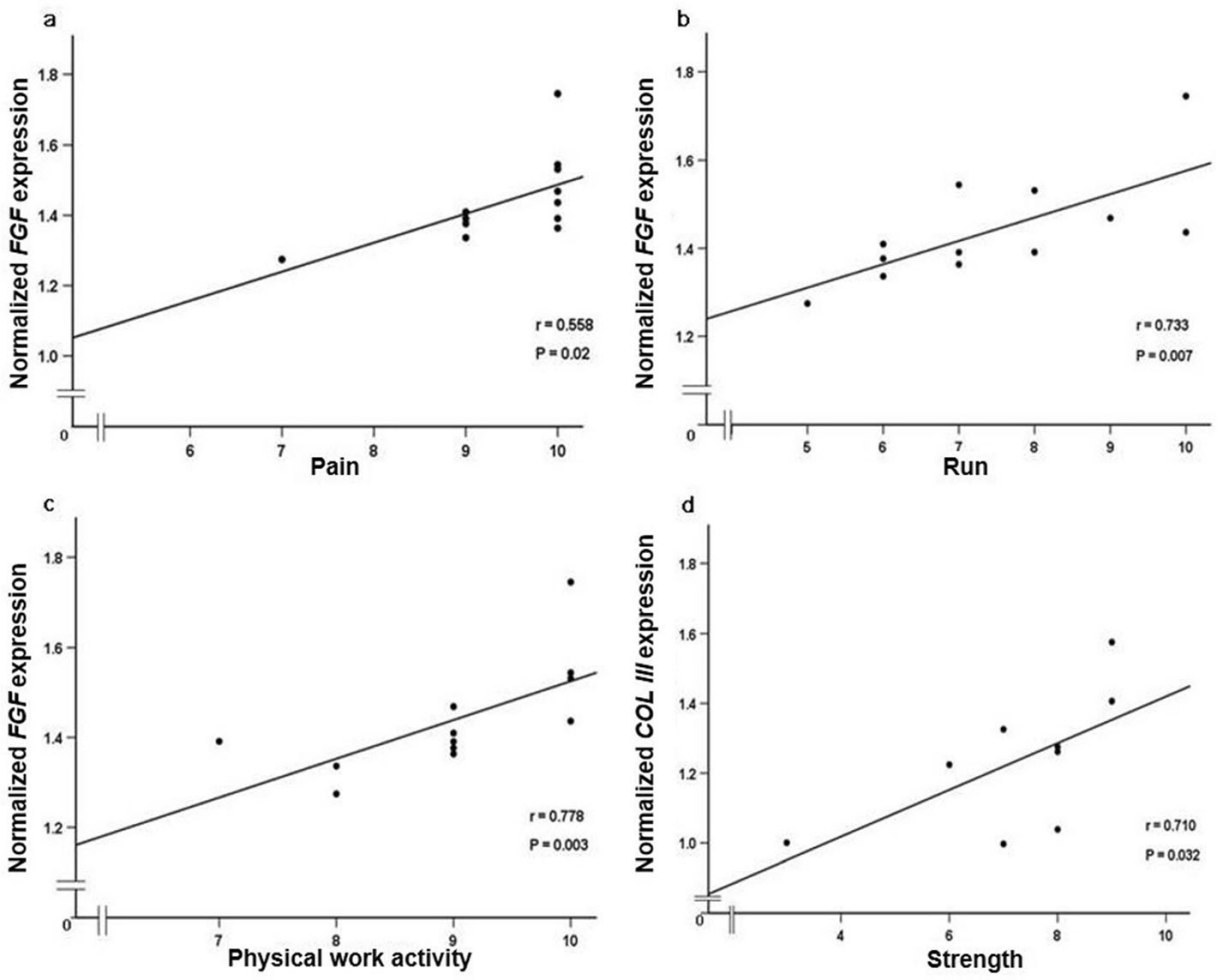
Associations among biomarker mRNA expression and ATRS sub-scales. Correlations between **a**) *FGF* gene expression and pain, **b**) *FGF* gene expression and run, **c**) *FGF* gene expression and physical work activity and, **d**) *COL III* gene expression and strength measured by Spearman’s rank correlation coefficient. ATRS sub-scales ranges from 0 to 10, with 10 = best outcome. *n* = 12

The associations between mRNA expression of *FGF*, *COL III*, *FN*, *COL I* and *MMP-9* in the injured tendon and ATRS and FAOS were studied. A positive association was observed between *FGF* mRNA expression and total ATRS (*r* = 0.711, *P* = 0.048) (Fig. 2) as well with ATR subscales of pain (*r* = 0.558, *P* = 0.02), less running limitations (*r* = 0.733, *P* = 0.007) and less loss in physical activity (*r* = 0.778, *P* = 0.003) (Fig. 3a-c). No associations were observed between total ATRS or ATR subscales and either of *COL III*, *FN*, *COL I* or *MMP-9* gene expression in the ATR patients except that of strength in the tendon which was positively correlated with higher *COL III* mRNA (*r* = 0.710, *P* = 0.032) expression in the injured tendon (Fig. 3d). No effect of age, gender, BMI or TTS was noted for any of the associations.

No associations were observed between any of the FAOS categories and *FN*, *COLIII*, *FN*, *COLI* or *MMP-9* gene expression in the ATR patients.

### Association between gene expression and HRT

**Fig. 4:**
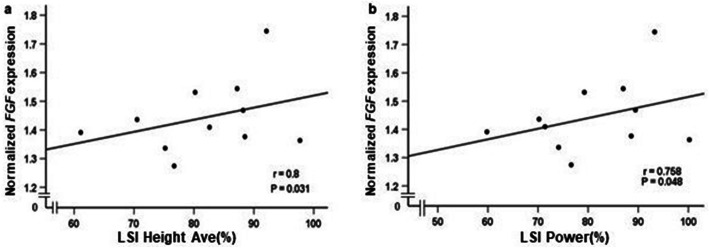
Associations among biomarker gene expression and HRT categories. Correlations between (**a**) *FGF* gene expression and LSI average height and, **b** *FGF* gene expression and LSI power measured by Spearman’s rank correlation coefficient. HRT categories ranges from 0 to 100, with 100 = best outcome. *n* = 12

A positive association was observed between *FGF* gene expression in the injured tendon and LSI power (*r* = 0.758, *P* = 0.048) and LSI- average height (*r* = 0.8, *P* = 0.031) (Fig. 4a, b). No associations were found between repetitions or total work and either of *COL III*, *FN*, *COL I* or *MMP-9* gene expression in the ATR patients.

### Associations among gene expression

**Fig. 5:**
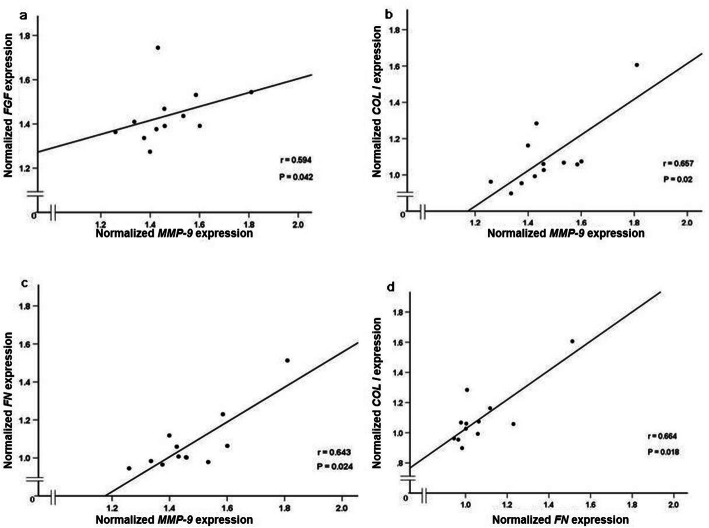
Associations among gene expression of **a** *MMP-9* and *FGF*, **b** *MMP-9* and *COL I*, **c** *MMP-9* and *FN* and, **d** *FN* and *COL* measured by Spearman’s rank correlation coefficient. *n* = 12

A positive association was observed between *FGF* and *MMP-9* gene expression (r = 0.594, *P* = 0.042) (Fig. 5a). No associations were observed for *FGF* and *COL III*, *FN or COL I* gene expression. Moreover, higher *MMP-9* was found to have a positive association with higher *COL I* (*r* = 0.657, *P* = 0.02) (Fig. 5b) and *FN* (*r* = 0.643, *P* = 0.024) gene expression (Fig. 5c). No association was observed among *MMP-9* and *COL III* mRNA levels. Additionally, higher *COL I* was positively associated with more *FN* mRNA expression (*r* = 0.664, *P* = 0.018) (Fig. 5d). No effect of age, gender, BMI or TTS was apparent for any of the associations. No associations were observed between *COL I* and *FGF* or *COL III* gene expression, and between *FN* and *FGF* or *COL III* gene expression.

### Histological assessment

**Fig. 6:**
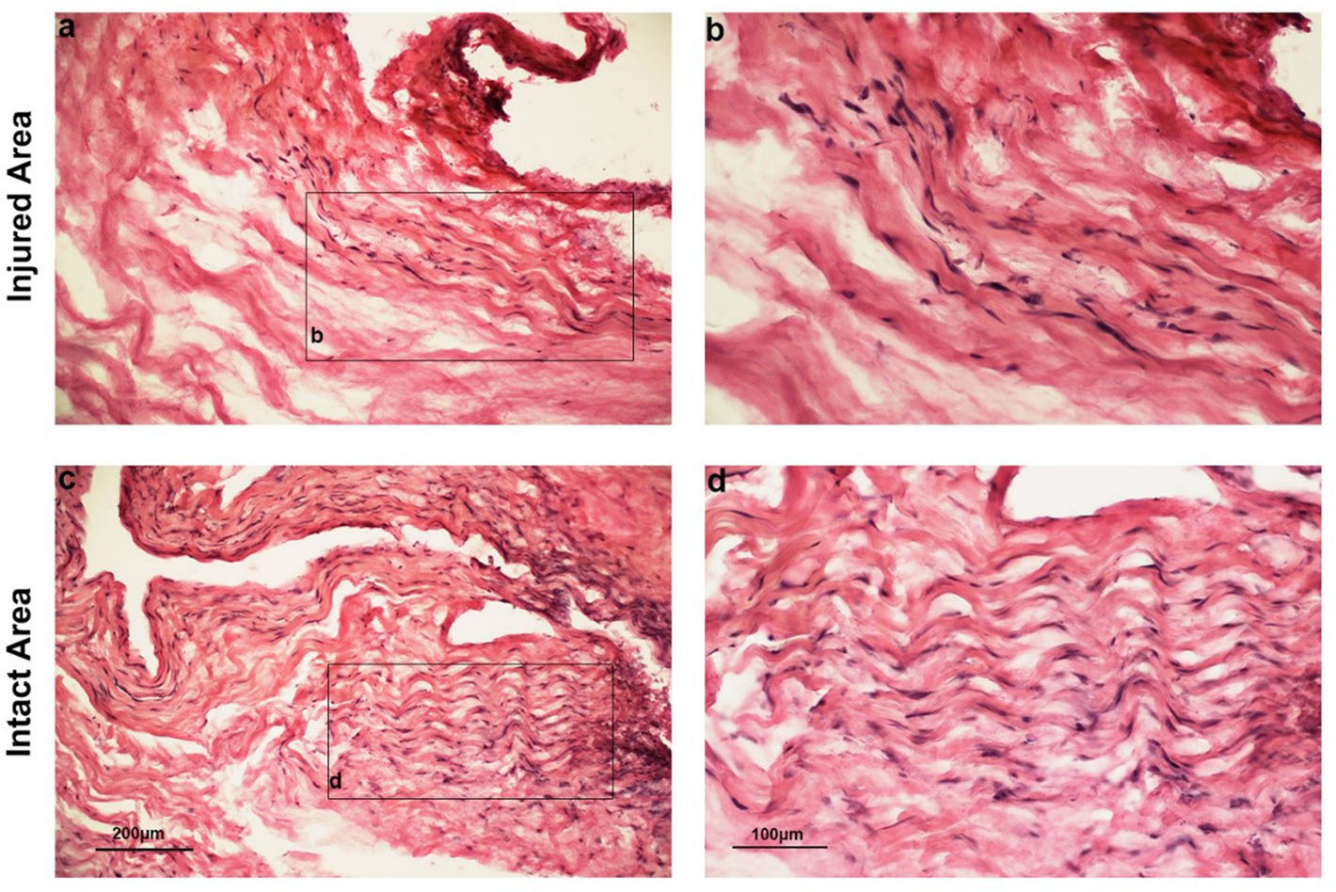
Photomicrographs of the injured and intact Achilles tendon stained with haematoxylin and eosin (**a-d**). All images are representatives from 5 patients. Original magnification is 10x (**a** and **c**) or 20X (**b** and **d**)

Our histological analysis did not detect any obvious differences in protein amount of *FGF*, *COL III*, *FN*, *COL I* and *MMP-9* between the injured and intact areas of the ruptured tendons, which was in line with the findings of the gene expression analysis. However, tendon collagen fibers were continuous and aligned in a parallel manner in intact areas, while disordered and broken fascicles were observed in the injured areas of the tendon at the time of surgery (Fig. 6a-d).

### Localization and expression of biomarkers

Overall, the immunohistochemical analysis of biomarkers at the protein level confirmed our finding of gene expression. However, a differential expression pattern and localization was observed for all studied biomarkers.

#### FGF

**Fig. 7:**
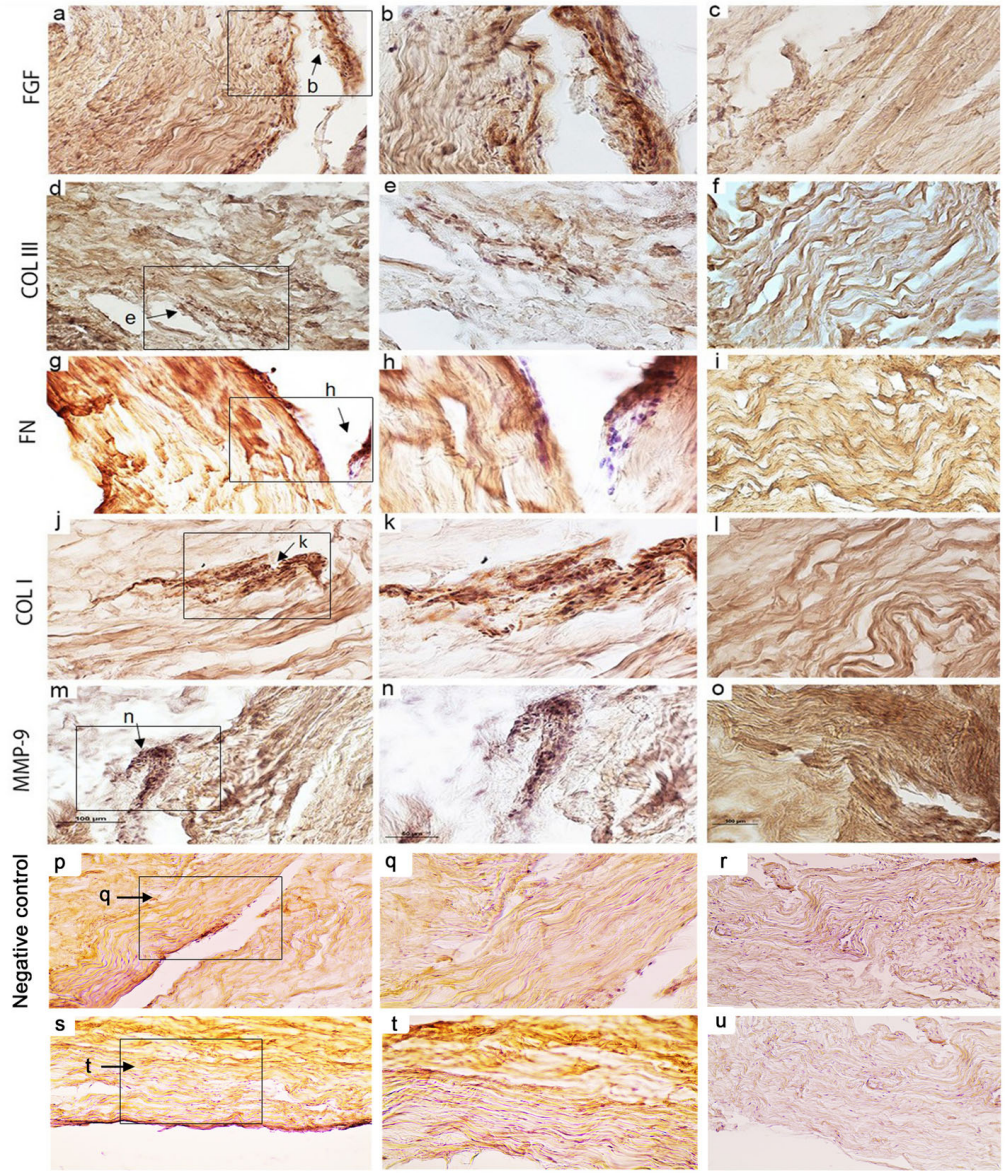
Protein expression of FGF (**a-c**), COL III (**d-f**), FN (**g-i**), COL I (**j-l**), MMP-9 (**m-o**), negative control for horse anti mouse (**p-r**), and goat anti rabbit (**s-u**) secondary antibodies in Achilles tendon tissue biopsies. Light microscope images are representative of 5 patients. The first and third columns displays 20x magnification, while the second column depict 40x magnification from the black rectangles in the first column. The first 2 columns display the injured side while the third column shows the intact area

FGF was expressed dispersed in both the intrafascicular tendon matrix and in the surrounding interfascicular connective tissues (Fig. 7a-c). In intact tendon, FGF was expressed mostly in the interfascicular matrix (Fig. 7c), while in the injured tendon, FGF was predominantly expressed close to blood vessels in the interfascicular connective tissues, but also observed in the intrafascicular matrix (Fig. 7a-b).

#### COL III

COL III was expressed extensively in both intrafascicular matrix and interfascicular connective tissues (Fig. 7d-f). In the intact tendon areas, COL III protein expression was found in the intrafascicular matrix (Fig. 7f) while in the injured tendon, COL III expression was observed in the tendon matrix as well in the interfascicular connective tissues (Fig. 7d-e).

#### FN

In intact tendon, FN expression was observed in the interfascicular matrix (Fig. 7g-h) while strong FN staining was observed both in intrafascicular matrix and in interfascicular connective tissues in the injured tendon areas (Fig. 7g-h).

#### Col I

Generally, COL I was expressed in both interfascicular connective tissues and intrafascicular matrix in biopsies (Fig. 7j-l). In intact areas, COL I was expressed in the intrafascicular matrix (Fig. 7l), while in the injured areas, COL I expressed both in the interfascicular connective tissues and the intrafascicular matrix (Fig. 7j-k).

#### MMP-9

In the intact tendon weak MMP-9 staining was found in the intrafascicular matrix (Fig. 7o). However, in the injured areas, *MMP-9* expression was mainly seen in the interfascicular connective tissues observed close to blood vessels (Fig. 7m-n).

## Discussion

To be best of our knowledge, the results of this study indicate for the first time that *FGF* gene expression in surgical tendon biopsies could be of importance as predictors to assist evaluation of ATR patient outcome 1 year post surgery. We found that higher *FGF* mRNA expression was correlated to improved patient outcome: less pain, less running limitations, less loss in physical work activity, more heel-rise height and more heel-rise power. Moreover, higher *COL III* gene expression was associated with increased tendon strength. Taken together, our results demonstrate that biomarker gene expression can be associated with long term patient-reported outcome in ATR patients.

The most important finding was that higher *FGF* gene expression was positively correlated to better patient-reported outcome 1 year after ATR, which could suggest an overall improved tendon healing process. The positive association between *FGF* mRNA expression and functional outcome strengthens our finding of improved tendon repair. Tendon healing is influenced by numerous processes such as cellular migration and proliferation, tissue remodeling, and synthesis of ECM, all these stages are orchestrated by variety of biomarkers [14, 16]. FGF is a multifunctional biomarker with several positive influences on tendon healing that have been demonstrated in previous studies [23, 39]. FGF initiates and stimulates the proliferation and migration of fibroblasts, i.e. in tendon so called tenoblasts, which are the important cells synthesizing new collagens. Our finding of higher FGF expression associated with better patient-reported outcome may be related to increased collagen production. In addition, a previous study showed that FGF can influence stem cells to express tendon-related genes, such as Collagen type I and III [13]. The increased FGF expression as observed in the present study, in injured Achilles tendon, presumably activates the production of COL I and III to improve the coding process of collagens, and also regulates the balance between ECM degradation and synthesis, leading to improved healing outcome. Moreover, FGF has been reported to strengthen the biomechanical properties in regenerative tendon by up-regulating the production of COL I and III [7]. Additionally, FGF has been identified to increase tendon healing by stimulating the orientation of collagen fibers and improving the biomechanical properties in a rabbit model of tendon healing [27]. No associations were found between the gene expression of the other four biomarkers examined and ATRS, indicating that FGF has a more essential role in long-term healing than the other biomarkers.

Increased *FGF* gene expression was associated with ATRS subscales of decreased pain, less running limitation and less loss in physical activity. Limitations in motion and loss of physical activity are two essential factors, which impact the quality of daily life for ATR patients after surgery. Pain may be one of the most important issues for ATR patients during the healing process. As reported previously, 44% of ATR patients exhibited pain in their injured leg 1 year after surgery [37], which impacts most of their daily life. Patients with ATR exhibit limitations in running, jumping, walking and loss of physical activity as well [11, 15, 26]. Pain is the main factor regulating the physical activity level in ATR patients. This may be the explanation for the simultaneous improvement in pain, running limitations and loss in physical activity as observed in the present study. Our study showed that higher *FGF* gene expression in human tendon was positively correlated to less pain, less running limitations and less loss in physical work activity. The comprehensive influence of FGF on the long-term patient-reported outcome reflects a multifunctional importance of FGF as a biomarker in tendon healing.

The second most important finding of this study was that higher *COL III* gene expression was associated with increased tendon strength. The healing process after ATR is slow and the content of collagens is changing during the initial inflammatory to later remodeling phase [3]. Tendons are mainly composed of collagen type I and III. COL I is the predominant type in normal tendon and make up bundles of thick fibrils [19], which maintain the tensile stiffness of the tendon. In contrast, COL III produces loosely packed bundles of thin fibers, so called scar tissue [8]. COL III is widely expressed among COL I bundles and is more flexible than COL I [35]. Conceivably, the increased *COL III* gene expression reflects more collagen type III production in the tendon at the time of surgery, which was at a mean of 3 days post rupture, i.e. early healing. We therefore suggest that the *COL III* gene expression is reflective of granulation/scar tissue repair in the early healing phase before the shift to COL I production has taken place.

Another interesting finding of our study was the positive association between *FGF* and *MMP-9* at the mRNA level. Earlier studies have reported on the interaction of *FGF* and *MMPs* in modulating tissue repair [20, 40]. In addition, FGF has been reported to facilitate the ECM remodeling and turnover by regulating the activation and synthesis of MMPs in mouse tendon repair [17]. Taken together, the results of the present study strengthen the previous observations of a strong biomodulatory connection between FGF and MMPs during tendon healing.

Our observation of *MMP-9* mRNA levels being positively associated with *COL I* and *FN* gene expression are in line with earlier findings. Increased MMP-9 and COL I protein expression have previously been reported in relation to the early healing process [24]. We further observed associations between gene expression of *COL I* and *FN* in injured Achilles tendon, which may seem logical based on their interaction in the healing process. We did, however, not find any association between *MMP-9*, *COL I*, *FN* and patient outcome. The reasons for these three genes not being correlated with patient outcome may be due to factors such as the timing of biopsy taking or to the fact that they do not have the same strong influence as *FGF* on patient outcome.

A potential limitation of this study is that we could not extract RNA from all studied subjects included in the study due to technical problems. The association between mRNA and outcome is not equal to an association between the protein and outcome, since transcriptional or post-translational regulation may change the protein expression. Based on limited number of samples, we did not fully explore the effects of age, sex, BMI or TTS on the associations among gene expression and ATRS subscale. However, the effects of age, sex, BMI and TTS were considered in the statistical analyses. Therefore, the identified associations should be considered as exploratory with the aim to identify promising therapeutic targets and need to be replicated in other larger studies.

## Conclusion

Taken together, our findings provide evidence that FGF expression in tendon biopsies of patients with acute ATR, can be used as predictor for prognostic evaluation of patient outcome, one-year post-surgery. Our findings also provide a foundation for the future development of FGF based novel therapies to improve healing after connective tissue injuries.
